# Lower Urinary Tract Symptoms in Uterine Myoma: A Systematic Review and Meta-Analysis

**DOI:** 10.3390/medicina61050890

**Published:** 2025-05-14

**Authors:** Lek-Hong Tan, Li-Hsien Tsai

**Affiliations:** Department of Urology, China Medical University Hospital, Taichung 404327, Taiwan; lekhongtan0810@gmail.com

**Keywords:** uterine myoma, lower urinary tract symptoms (LUTS), urinary incontinence, fibroids, pelvic floor dysfunction, hysterectomy, systematic review and meta-analysis

## Abstract

*Background and Objectives*: Lower urinary tract symptoms (LUTSs) are prevalent among patients with uterine myoma (UM); however, these health issues have not been systematically evaluated. To address this research gap, this systematic review and meta-analysis synthesizes existing findings on the prevalence estimates and odds ratios for LUTSs in patients with UM. *Materials and Methods*: A systematic literature search using PubMed and Embase was conducted for articles published between 1 January 2000 and 24 September 2023. The search and review processes followed the PRISMA and MOOSE guidelines. This study was registered in PROSPERO (CRD42023474156). Data on the prevalence and odds ratios of LUTSs—including storage symptoms (frequency, urgency, nocturia), voiding symptoms, and urinary incontinence (UI) subtypes such as stress incontinence (SUI), urgency urinary incontinence (UUI), and mixed urinary incontinence (MUI)—were extracted. Pooled prevalence estimates and odds ratios were calculated using random-effects meta-analysis. Subgroup analyses and univariate meta-regression were conducted to examine associations with age, BMI, parity, WHO region, and risk of bias. The impact of UM size was assessed using standardized mean differences. *Results*: Of the 572 articles screened, 20 met the inclusion criteria. The overall pooled prevalence of LUTSs in UM patients was 49% (95% CI, 26–72%), with substantial heterogeneity across studies (I^2^ = 99.8%). The pooled prevalence for urinary frequency, urgency, nocturia, voiding dysfunction, and overall UI, SUI, UUI, and MUI ranged from 15% to 54%. SUI and UUI were significantly associated with UM (OR = 2.0, 95% CI: 1.2–3.3; OR = 1.5, 95% CI: 1.1–2.0, respectively). Hysterectomy was not associated with an improvement in overactive bladder (OAB) symptoms (OR = 1.9, 95% CI: 0.6–5.7). A larger UM size was not linked to worsening LUTS. Fourteen studies (70%) had some concerns about the risk of bias, while six studies (30%) had a low risk of bias. Egger’s test showed no significant publication bias (*p* = 0.19). *Conclusions*: Approximately half of patients with UM experience LUTSs or UI. The findings emphasize the need to consider urinary symptoms in UM management. Further research is warranted to reduce heterogeneity and explore treatment-specific outcomes.

## 1. Introduction

Uterine myomas (UMs), benign tumors originating from the smooth muscle cells of the uterine wall, represent a common gynecological condition affecting a substantial proportion of women during their reproductive years [1,2,3,4]. The prevalence of uterine myomas is wide-ranging, with estimates varying from 4.5% to 80% depending on the demographic characteristics of the population under study [4]. Traditionally, these tumors have been primarily associated with reproductive health concerns, including infertility, pregnancy complications, and abnormal bleeding [4]. However, emerging research suggests a significant and often overlooked link between uterine myomas and lower urinary tract symptoms (LUTSs) [5].

LUTSs encompass a diverse range of bothersome clinical manifestations, such as increased urinary frequency, urgency, nocturia, and urinary incontinence, collectively contributing to a considerable decline in the quality of life of affected individuals [6,7]. The intricate relationship between uterine myomas and LUTSs involves multifaceted mechanisms, including mechanical compression, hormonal influences, and potential neurogenic factors [8,9,10]. While individual studies have explored this association, a comprehensive synthesis of the existing evidence through a systematic review and meta-analysis is paramount to discerning the nuanced interplay between uterine myomas and lower urinary tract symptoms.

This research aims to address a significant gap in the existing literature by conducting a rigorous examination of available studies investigating the relationship between uterine myomas and lower urinary tract symptoms (LUTSs). Through a systematic review and meta-analysis, our objective is to not only clarify the prevalence of LUTSs in women with uterine myomas but also to identify the specific association between uterine myomas and these lower urinary tract symptoms. Additionally, our study seeks to evaluate the impact of various treatment modalities, where data can be synthesized, on alleviating lower urinary tract symptoms in this specific population. This synthesis of evidence holds critical importance in informing clinicians, aiding in treatment decisions, and ultimately enhancing overall care and outcomes for women dealing with uterine myomas and associated lower urinary tract symptoms.

## 2. Methods

### 2.1. Search Strategy and Selection Criteria

Our methodological approach to conducting this systematic review and meta-analysis aligns with the guidelines set forth in the Preferred Reporting Items for Systematic Reviews and Meta-Analyses (PRISMA) Statement and adheres to the Meta-Analysis of Observational Studies in Epidemiology (MOOSE) criteria [11,12]. The exploration was centered on epidemiologic studies investigating the prevalence and odds ratios of LUTSs in individuals with UM. LUTSs encompassed storage symptoms (frequency, urgency, and nocturia), voiding symptoms, and urinary incontinence, including stress incontinence, urinary urgency incontinence, and mixed urinary incontinence. This comprehensive search spanned the period from 1 January 2000 to 24 September 2023, encompassing PubMed/MEDLINE and Embase databases. The search strategy utilized Medical Subject Headings terms and specific keywords, which are detailed in Table S1.

Inclusion criteria encompassed the following: (1) observational studies; (2) studies involving UM patients with or without a comparator group; and (3) studies presenting quantitative data on the prevalence and odds ratios of LUTSs. Language restrictions were not applied, and translations, if needed, were facilitated using Google Translate and ChatGPT 3.5.

Post-duplicate removal, we excluded studies lacking original data (reviews, editorials, letters, case reports, and commentaries), studies with fewer than 10 participants, and those lacking relevant endpoints. Conference abstracts were also omitted. A rigorous assessment of the remaining articles involved a thorough examination of full texts, excluding studies without extractable data (e.g., lacking measured point prevalence, odds ratios, or data for calculating the prevalence or odds ratio for LUTSs in UM). Duplicate populations were identified and excluded. This study adhered to the PROSPERO-registered protocol (www.crd.york.ac.uk/PROSPERO; protocol number CRD42023474156).

### 2.2. Definition of Lower Urinary Tract Symptoms

LUTSs comprise a spectrum of manifestations involving storage, voiding, and post-micturition symptoms [13]. Storage symptoms encompass increased frequency, urgency, nocturia, and UI, which can manifest as SUI, UUI, or MUI. Voiding symptoms involve hesitancy, intermittency, a slow urinary stream, straining, and the occurrence of the splitting or spraying of the urinary stream, along with terminal dribble. Post-micturition symptoms include sensations of post-void dribbling and perception of incomplete bladder emptying.

In studies where multiple subtypes of LUTSs were reported within the same population, the symptom with the highest prevalence estimate was chosen to represent the prevalence of LUTSs in that particular population. This selection was made for the purpose of calculating the pooled overall prevalence of LUTSs in the subsequent meta-analysis.

### 2.3. Definition of Overactive Bladder

OAB, which constitutes another subgroup of LUTSs, is characterized by urinary urgency and is often accompanied by urinary frequency and nocturia, with or without urgency urinary incontinence [13,14,15]. In this study, patients exhibiting urinary urgency, UUI, and diagnosed with OAB were incorporated into the OAB subgroup for meta-analysis.

### 2.4. Data Abstraction

The assessment of references by titles and abstracts was conducted independently by two authors (L.H. Tan and L.H. Tsai), with the retrieval of relevant full texts for further examination. In instances where articles were inaccessible, efforts were made to establish contact with the respective authors. The systematic extraction of information, encompassing study characteristics and prevalence estimates related to LUTSs in UM, was carried out, and the results are detailed in Table S2. In cases where multiple studies reported on the same population, preference was given to the study with the largest sample size and the most informative data. Discrepancies among reviewers were resolved through consensus.

For studies not directly providing prevalence estimates or odds ratios, calculations were derived from extractable information. Pooled odds ratios were computed to compare patients with UM who underwent total hysterectomy with those who did not, assessing the impact on OAB. Additionally, pooled standardized mean differences (SMDs) were estimated to analyze the effect of UM size (small versus large) on LUTSs.

To assess study quality, a modified risk-of-bias tool tailored for prevalence studies was employed based on the validated framework by Hoy et al. (2012) [16]. This tool comprises ten items covering four domains: external validity (selection and nonresponse bias), internal validity related to measurement bias, and internal validity related to analysis. Each item was rated as having a low or high risk of bias, with insufficient information conservatively rated as high risk. A summary judgment categorized each study as having an overall low risk, some concerns (moderate risk), or a high risk of bias. The detailed criteria for each item are provided in Supplementary Table S3.

Utilizing the robvis visualization tool [17], we generated “traffic light” plots illustrating domain-level assessments for individual results. Additionally, weighted bar plots were crafted to depict the distribution of risk-of-bias judgments within each bias domain (Figure S1).

### 2.5. Data Analysis

Pooled prevalence rates and odds ratios were computed through a random-effects meta-analysis, with stratification based on subtypes of LUTSs. The Comprehensive Meta-Analysis (CMA) software, version 4 (Biostat, Englewood, NJ, USA), was employed for the entirety of the analyses. Subgroup investigations were undertaken to assess potential sources of heterogeneity, focusing on prevalence estimates related to LUTSs in particular. Covariates subjected to analysis included the (1) mean age; (2) body mass index (BMI); (3) parity; (4) World Health Organization (WHO) region; and (5) risk of bias. Univariate meta-regression plots were generated to illustrate the correlations between pooled estimates and mean age, BMI, and parity. Each study was represented by circles in these plots.

Substantial heterogeneity, as indicated by I^2^ tests (values exceeding 50%), was taken into account. Funnel plots and Egger’s test were employed to scrutinize potential publication bias. The threshold for statistical significance was set at *p*-values < 0.05, except in the case of determining publication bias, where a significance level of *p* < 0.10 was considered.

## 3. Result

### 3.1. Overview of Enrolled Studies

**Figure 1:**
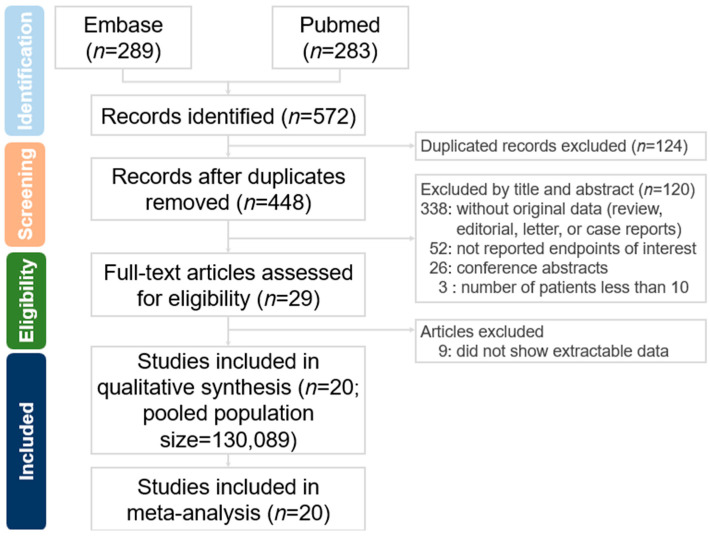
Flow chart for full-text screening.

We identified 572 studies in the secondary databases, and 29 studies were eligible for full-text screening (Figure 1). We identified 20 articles that met the inclusion criteria and provided prevalence and odd ratio data for LUTSs from the eligible articles. Of the 130,089 participants from the 20 included studies, 126,318 patients were diagnosed with uterine myoma (UM), and the rest were treated as controls who did not have UM. Five articles (*n* = 65,014) included data on LUTSs after hysterectomy, with one article including data on post-hysterectomy LUTSs only. There was no available post-UM treatment LUTS data (such as myomectomy, radiofrequency ablation, High-Intensity-Focused Ultrasound, embolization, or pharmacological treatment) in the included studies that were able to be synthesized for meta-analysis. There were only three included studies demonstrating synthesizable data regarding the impact of UM size on LUTSs for meta-analysis. There was no synthesized data regarding the impact of the UM position on LUTSs in the included studies for meta-analysis.

### 3.2. Studies on LUTSs in UM Without Hysterectomy

In a total of 19 studies that reported on UM without or before hysterectomy, the prevalence of LUTSs was estimated. For storage symptoms in UM, nine studies reported data on frequency (*n* = 1134), five studies reported nocturia data (*n* = 816), and three studies reported urgency data (*n* = 4083). For urinary incontinence in UM, nine studies reported SUI data (*n* = 1700), six studies reported UUI data (*n* = 1081), two studies reported MUI data (*n* = 701), and five studies reported UI data without specific subtypes (*n* = 7551). Five studies report data on voiding symptoms in UM (*n* = 435). Eleven studies reported OAB (*n* = 121,914). Three studies reported odds ratio data for SUI; two studies reported odds ratio data for MUI; and two studies reported odds ratio data for UUI. However, none of the studies included in the analysis reported post-micturition symptoms. This likely reflects a methodological limitation, as most studies employed symptom assessment tools or questionnaires that did not include post-micturition domains. The overall mean age of all enrolled UM patients with LUTSs was 42.5 years (with a standard deviation of 2.8), overall parity was 1.9 (with a standard deviation of 0.5), and overall BMI was 27.3 kg/m^2^ (with a standard deviation of 3.5). All the studies were observational studies, either retrospective cohort studies or cross-sectional studies that used the consecutive sampling method. The geographical distribution of study populations was assessed across three continents, categorized by WHO regions. Specifically, ten studies were conducted in the European region, eight in the region of the Americas, and one in the Western Pacific region, while no relevant studies were identified in the African region, South-East Asian region, or Eastern Mediterranean region.

### 3.3. Studies on LUTSs in UM After Hysterectomy

The prevalence of LUTSs after hysterectomy was also estimated in five studies, in which two studies reported data on frequency (*n* = 320); two studies reported data on urgency (*n* = 3778); two studies reported SUI data (*n* = 320); two studies reported UUI data (*n* = 320); two studies reported UI data without specific subtypes (*n* = 6499); two studies reported the data of voiding dysfunction in UM (*n* = 320); and four studies reported OAB (*n* = 62,133). Two studies reported odds ratio data for comparing OAB in UM with and without hysterectomy. The one article that included only post-hysterectomy LUTS data was a retrospective study using the consecutive sampling method, with mean age of 44.1 (with a standard deviation of 7.2), and was conducted in the Western Pacific region.

### 3.4. Risk Assessment of Bias

Summaries of the risks of bias of the selected studies for LUTSs in UM are presented in Figure S1. Of the 20 included studies on LUTSs, 17 (85.0%) studies were judged as having a low risk, and 3 (15.0%) studies were deemed to have some concerns or a moderate risk.

### 3.5. Meta-Analysis

**Figure 2:**
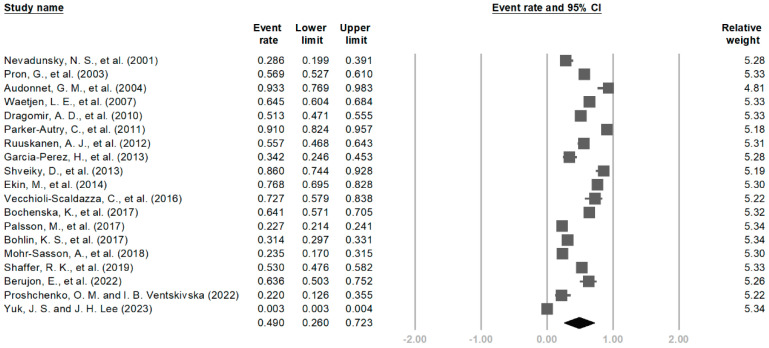
The overall pooled prevalence of LUTSs among patients with UM and without a hysterectomy [10,18,19,20,21,22,23,24,25,26,27,28,29,30,31,32,33,34,35,36]. *Note: Squares represent individual study estimates, and diamond represent the pooled summary effect*.

The overall pooled prevalence of LUTSs among patients with UM without a hysterectomy (Figure 2) was 49.0% (95% CI, 26.0–72.3%), with a high degree of heterogeneity (I^2^ = 99.8%). The prevalence estimates of LUTSs ranged from 0.3 to 91.0%. The pooled prevalence of urinary frequency was 54.2% (95% CI, 42.6–65.3%), 39.5% (95% CI, 20.5–62.4%) for urgency, 44.6% (95% CI, 27.6–63.0%) for nocturia, 45.1% (95% CI, 30.5–60.5%) for overall UI, 49.0% (95% CI, 41.5–56.6%) for SUI, 21.8% (95% CI, 10.1–40.9%) for UUI, 15.2% (95% CI, 4.4–41.1%) for MUI, 34.3% (95% CI, 18.7–54.2%) for voiding symptoms, and 24.2% (95% CI, 5.6–63.2%) for OAB (Figure S2). The prevalence of LUTSs in UM after a hysterectomy was comparable to UM without a hysterectomy, which is shown in Figure S3.

Both SUI and UUI were associated with uterine myoma when comparing the UM arm to the non-UM arm [the pooled odds ratio was 2.0 (95% CI, 1.2–3.3, *p* = 0.006) and 1.5 (pooled odds ratio: 95% CI, 1.1–2.0, *p* = 0.014), respectively]. However, for MUI, no statistical significance was achieved [1.9 (95% CI, 0.9–3.9, *p* = 0.07)] (Figure S4). A larger UM size was not associated with worsening LUTSs across all subtypes (Figure S5). A hysterectomy was not associated with an improvement in OABs in patients with UM compared to those who did not receive a hysterectomy [pooled odds ratio: 1.9 (95% CI, 0.6–5.7, *p* = 0.251)] (Figure S6).

**Figure 3:**
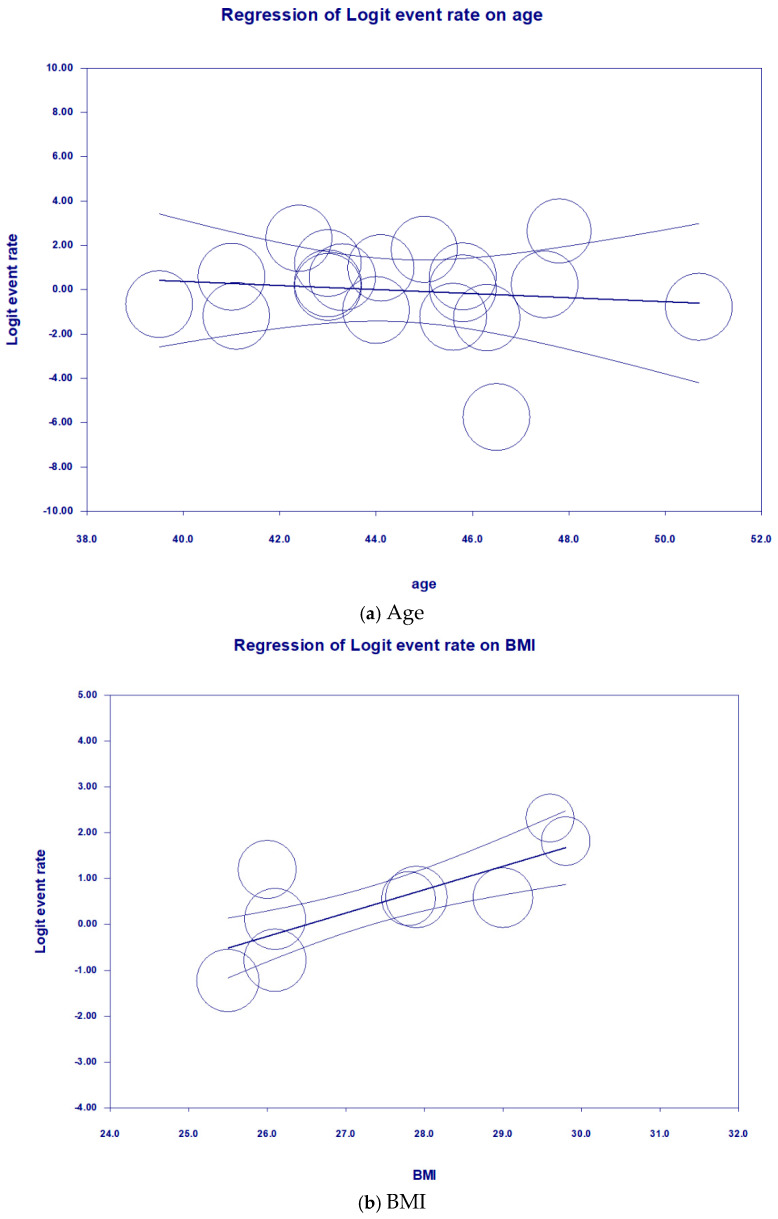

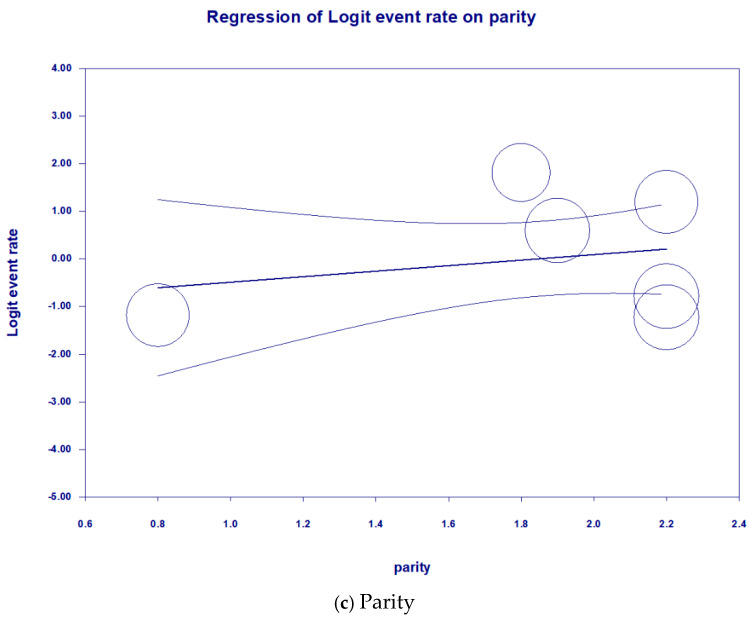
Univariate random-effects meta-regression for the prevalence of LUTSs according to age categories, parity, and BMI. *Note: Circles represent individual studies, with size proportional to study weight. The central line indicates the fitted meta-regression line, and the outer lines represent the 95% confidence interval*.

**Figure 4:**
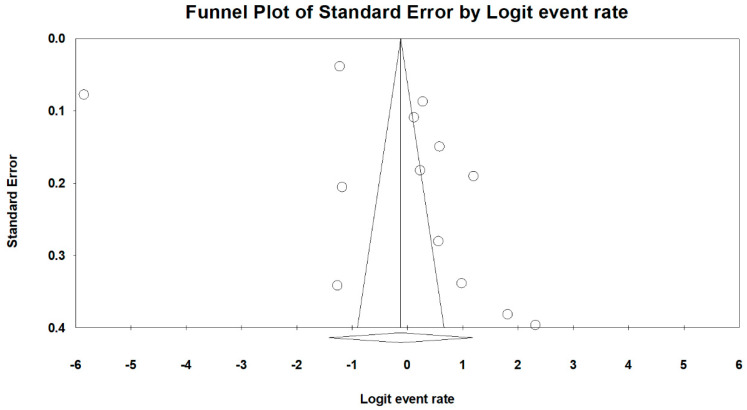
A funnel plot of the prevalence of LUTSs in patients with UM. *Note: Circles represent individual studies. The vertical line indicates the overall pooled logit event rate. Diagonal lines represent the 95% pseudo confidence limits. The diamond represents the adjusted effect size and confidence interval estimated by the Trim-and-Fill method*.

The univariate random-effects meta-regression (Table 1 and Figure 3) showed that the prevalence estimates of LUTSs in UM are significantly associated with an increase in BMI (*p* < 0.05), accounting for 68% of the variability in effect sizes. On the other hand, neither an increase in age (*p* = 0.67) nor parity (*p* = 0.37) was associated with the prevalence of LUTSs and, therefore, could not explain the variability in effect sizes. Risks of bias did not alter the prevalence estimates of LUTSs in UM (*p* = 0.89). The prevalence estimate of LUTSs in UM in the European region was similar to that in the region of the Americas (*p* = 0.62). However, the univariate random-effects meta-regression showed that the result from the Western Pacific region contributed a significant amount of variance (*p* < 0.001). Egger’s test revealed no significant publication bias regarding the overall prevalence of LUTSs (*p* = 0.19) (Figure 4).

## 4. Discussion

### 4.1. Prevalence of LUTSs in UM

The meta-analysis revealed a substantial prevalence of LUTSs among patients with UM, with an overall pooled prevalence of 49.0%. This high prevalence underscores the clinical significance of considering LUTSs in the management of UM. Storage symptoms, including urinary frequency, urgency, and nocturia, were prevalent among UM patients, indicating a significant burden on quality of life. Additionally, urinary incontinence, including stress urinary incontinence (SUI), urgency urinary incontinence (UUI), and mixed urinary incontinence (MUI), was common, further highlighting the impact of UM on urinary function. Voiding symptoms and OAB were also prevalent, contributing to the overall symptomatology experienced by UM patients. The prevalence estimate of LUTSs in UM in the European region was comparable to that in the region of the Americas.

### 4.2. Association with Hysterectomy and Other Interventions

Contrary to expectations, hysterectomy did not significantly alleviate LUTSs in UM patients. The comparable prevalence of LUTSs in UM before and after hysterectomy suggests that surgical intervention may not effectively address urinary symptoms in this population. Additionally, the lack of improvement in OAB symptoms post-hysterectomy emphasizes the complex etiology of LUTSs in UM patients, warranting further investigation into alternative management strategies.

A large Swedish epidemiological study with a mean follow-up time of 12 years showed that hysterectomy increased the risk of subsequent surgery for stress urinary incontinence, with a hazard ratio of 2.4. The authors of that study concluded that the most plausible explanation for the increased hazard ratio was the effect of the surgical trauma inflicted during hysterectomy [37]. Consistently, a Danish nationwide study involving over 83,000 women who underwent hysterectomy for benign indications reported that these women had more than double the risk of requiring SUI surgery compared to matched controls (adjusted hazard ratio [aHR] 2.6; 95% confidence interval [CI], 2.4–2.8). This elevated risk persisted even after excluding vaginal hysterectomies (aHR 2.4; 95% CI, 2.3–2.6). Notably, the risk was particularly pronounced among women with a history of vaginal births; for instance, women with one vaginal birth who underwent hysterectomy had a 15-fold increased risk of SUI surgery compared to nulliparous women (aHR 15.1; 95% CI, 10.3–22.1) [38]. A study reported that the incidence rates of urinary bladder and ureteral injuries in patients undergoing total hysterectomy were 1.1% and 1.9%, respectively [39]. Moreover, the surgical trauma caused when the uterus and cervix are severed from pelvic-floor-supportive tissues can affect neuroanatomical structures [40]. Hysterectomy could interfere with the intricate urethral sphincter mechanism by damaging distal branches of the pudendal nerves and inferior hypogastric plexus [41]. It might also result in changes to urethral and bladder neck support [42]. The acute pelvic-floor tissue trauma caused by hysterectomy has been presumed to result in adverse effects that can be chronic and progressive over time [37]. Therefore, the effect of hysterectomy on relieving LUTSs of UM may be offset by surgical factors. However, patients with a larger uterine size may experience a protective effect against LUTSs, including incontinence and urgency, after receiving a hysterectomy [29].

Uterine artery embolization (UAE) is an alternative option for fibroid treatment in women who wish to avoid surgery or when it is contraindicated. Few published studies have assessed LUTSs before and after UAE, but they have shown an improvement in LUTSs after the intervention [19,25]. One study proposed a theory that UAE helps improve LUTSs by addressing the hypervascularization of UM, which may cause symptoms such as shunting blood from the surrounding pelvic organs and creating a state of relative chronic hypoxia. By overcoming this shunt, embolization may improve bladder vascularization [43]. However, in most studies assessing UAE, LUTSs were assessed only as a secondary outcome, and the methods of assessment varied across different studies, so these studies were not included in this meta-analysis.

Currently, the literature regarding the effects of myomectomy on LUTSs is scarce. Most studies investigating UM surgery outcomes included myomectomy in the category of UM or fibroid surgery without subgroup analysis. Therefore, the effects of myomectomy on LUTSs remain inconclusive. Also, no study has investigated the effect of medication or hormonal therapy on UM patients on LUTSs.

This lack of synthesizable data on non-hysterectomy interventions is a significant limitation of our meta-analysis. It restricts our ability to evaluate and compare the effect of different treatment modalities on urinary symptoms. This gap highlights the need for future prospective studies with standardized LUTS assessment tools to explore the impact of myomectomy, UAE, and medical therapies on urinary outcomes in women with uterine myomas.

### 4.3. Impact of UM Characteristics

The meta-analysis found no significant association between UM size and worsening LUTSs, indicating that the size of the myoma may not be a primary determinant of urinary symptom severity. However, the position of the UM and uterine volume may be related to worsening LUTSs. A few studies showed that the presence of an anterior UM was significantly associated with higher urinary symptoms [10,33,44,45]. Additionally, women with moderate and severe urinary urgency had significantly larger uterine volumes [10]. It is important to note that while these findings suggest the potential role for myoma’s position—particularly regarding the anterior wall location—this factor was not included in our meta-analysis due to the limited number of studies reporting analyzable data on UM’s position.

Our study showed that stress urinary incontinence (SUI) and urgency urinary incontinence (UUI) have become significantly associated with UM. SUI may be caused by increased abdominal pressure leading to the forward movement of myomas, which “hit the bladder” and increase bladder pressure, causing urinary leakage [5]. A study hypothesized that vesical hyperactivity associated with myomas could be due to bladder compression increasing the proprioceptive signal, leading to urinary frequency and urgency [8]. On the other hand, the growth of leiomyomas in the broad ligament can result in the obstruction of the bladder outlet and paravaginal obstruction, resulting in urinary retention [46]. This insight can inform clinical decision-making regarding the evaluation and treatment of urinary symptoms in UM patients.

### 4.4. Factors Influencing the Prevalence of LUTSs

This study identified body mass index (BMI) as a significant factor influencing the prevalence of LUTSs in UM patients, with a higher BMI correlating with increased LUTS prevalence. This finding underscores the potential impact of lifestyle modifications and weight management strategies on mitigating urinary symptoms in UM patients. Several biological mechanisms may explain this association. Obesity is associated with increased intra-abdominal pressure, which can place stress on the bladder and pelvic floor structures, potentially exacerbating urinary symptoms [47]. It is also linked to systemic inflammation and vascular dysfunction, which may contribute to chronic pelvic ischemia—a condition shown in animal models to cause detrusor overactivity [48]. In addition, obesity can impair nitrergic signaling, as nitric oxide (NO) released from nitrergic nerve terminals is an essential nonadrenergic and noncholinergic neurotransmitter in the smooth muscle of the lower urinary tract, including the bladder, urethra, and ureter [49]. Reduced NO bioavailability has been associated with lower urinary tract dysfunction in other contexts, suggesting a plausible mechanism by which obesity may influence LUTSs. Beyond these mechanistic insights, clinical evidence from human studies has consistently shown that obesity is a major risk factor for pelvic floor dysfunction and urinary incontinence. Large-scale observational studies have demonstrated a positive association between higher BMI and both stress and urgency incontinence, as well as storage LUTSs [47]. Interventional studies further support this link, showing that weight reduction—whether through lifestyle changes or bariatric surgery—can lead to significant improvements in urinary symptoms and pelvic floor function [50]. Moreover, obesity has been identified as a factor that can significantly increase the complexity of surgical procedures [51]. However, weight loss, either by changing habits or through surgical intervention, leads to decreased SUI prevalence and severity [52,53].

Conversely, age and parity did not appear to significantly influence the prevalence of LUTSs in our study. One possible reason for this is that the age groups and parity of the populations in the studies included in the meta-analysis were quite similar; the average age was between 40 and 50 years old, and almost all of the population had an average parity of two. Therefore, these two factors did not contribute to the variability in effect sizes.

Although the meta-regression indicated that the Western Pacific region contributed significantly to heterogeneity, this finding must be interpreted with caution. It was based on a single study from this region, which reported an unusually low LUTS prevalence. This result may reflect study-specific factors rather than broader regional characteristics and, therefore, may not be representative of the overall population in the Western Pacific. The limited number of studies from several WHO regions further constrains the interpretability of geographic comparisons. Future research incorporating a more balanced regional distribution is needed to better understand potential geographic differences in LUTS prevalence among UM patients.

In addition to biological and demographic factors, psychosocial conditions in the workplace may also contribute to LUTS prevalence. Studies have shown that working women exposed to high emotional labor, job stress, and shift work are more likely to experience LUTSs, which can significantly reduce quality of life and work productivity. A Korean cross-sectional study found that job-related stress was associated with increased LUTSs and lower QoL among employed women [54]. Furthermore, a systematic review involving over 48,000 female workers indicated that LUTSs are associated with impaired productivity—particularly presenteeism—though not significantly with absenteeism [55]. These findings suggest that workplace psychosocial stressors are relevant to risk factors that should be considered in the broader context of LUTS management and prevention in working populations.

### 4.5. LUTSs in Malignant vs. Benign Uterine Tumors

Lower urinary tract symptoms (LUTSs) in malignant uterine tumors, such as endometrial cancer, often arise from mechanisms distinct from those in benign conditions like uterine fibroids. While fibroids typically cause LUTSs through the mechanical compression of the bladder, malignant tumors may lead to urinary symptoms due to local invasion, the disruption of pelvic anatomy, or the effects of cancer treatments. For instance, patients with endometrial cancer can present with difficulty urinating and pelvic pain, especially in advanced stages where the tumor invades adjacent structures [56]. Additionally, treatments like surgery and radiotherapy for gynecological malignancies have been associated with the increased prevalence of urinary incontinence and other pelvic floor dysfunctions [57]. In contrast, benign uterine tumors like fibroids primarily cause LUTSs through mass effect without tissue invasion, and symptoms may persist or recur depending on fibroid characteristics and treatment approaches [33].

### 4.6. Emerging Evidence from Recently Published Studies

Following peer review, we conducted an updated literature search extending to 31 December 2024 and identified three newly published studies relevant to LUTSs in patients with uterine myoma. A retrospective cohort study by Agu et al. reported that leiomyomas larger than 6 cm were significantly associated with an increased prevalence of LUTSs, including frequency and nocturia, with an odds ratio of 3.1 [58]. A population-based cohort study from South Korea by Yuk et al. demonstrated a modest but statistically significant association between abdominal hysterectomy for fibroids and the subsequent risk of anti-incontinence surgery, reinforcing concerns about long-term pelvic floor consequences [59]. Finally, a retrospective single-center study by Zhu et al. found that pelvic floor functional exercise following fibroid surgery significantly reduced LUTS incidence and improved pelvic floor muscle strength and quality of life [60]. These recent findings provide further support for the multifactorial nature of LUTSs in UM patients and highlight important avenues for future interventional and longitudinal research.

### 4.7. Limitations and Future Directions

While the systematic review and meta-analysis provide valuable insights into LUTS prevalence and associations in UM patients, several limitations should be acknowledged. The observational studies predominately included retrospective cohorts and cross-sectional designs, which limited the ability to draw causal inferences. Furthermore, many of the included studies did not adequately account for potential confounding variables, such as comorbidities (e.g., diabetes and hypertension), menopausal or hormonal status, and other lifestyle factors. Although a few studies reported adjusted odds ratios, the lack of consistent multivariable adjustment increases the risk of residual confounding and complicates the interpretation of associations between UM and LUTSs.

Additionally, the high heterogeneity among the included studies may limit the generalizability of the findings. The lack of standardized methodologies across the studies poses further challenges to data interpretation. One methodological limitation is our approach to estimating overall LUTS prevalence—specifically, in studies that reported multiple LUTS subtypes, where we selected the symptom with the highest prevalence to represent overall LUTSs. This may have led to an overestimation of the pooled prevalence, as it does not reflect the full distribution of coexisting symptoms.

Another key limitation is the lack of synthesizable data on interventions other than hysterectomy. Although several studies discussed treatments such as uterine artery embolization (UAE), myomectomy, or pharmacological therapies, most did not provide analyzable data on LUTS outcomes or used heterogeneous assessment methods. This limited our ability to evaluate and compare the effects of different treatment modalities on urinary symptoms in women with UM. Among these, UAE appears particularly promising, with some studies reporting improvements in LUTSs following the procedure. However, due to heterogeneity in the outcome measurements and study design, UAE outcomes could not be included in the current meta-analysis. Future research should specifically explore the role of UAE using standardized LUTS assessment tools and consistent follow-up protocols to determine its therapeutic potential.

Furthermore, the conclusion that hysterectomy does not significantly improve LUTSs was based on a limited number of studies (*n* = 5), and one of these studies—originating from the Western Pacific region—reported an unusually low LUTS prevalence, which may not be representative. As such, this conclusion should be interpreted cautiously and may not be generalizable across different populations.

Additionally, this review excluded non-English language publications and unpublished data such as conference abstracts or theses. Although Egger’s test did not reveal significant publication bias, we acknowledge that this test has limited power, particularly with small sample sizes. Therefore, the exclusion of non-peer-reviewed and non-English literature could still introduce selection or language bias. Future systematic reviews may consider incorporating gray literature and studies published in other languages to enhance comprehensiveness and reduce the risk of publication bias.

Future research should aim to address these limitations through prospective study designs, including well-designed randomized controlled trials comparing different treatment modalities (e.g., hysterectomy, UAE, myomectomy, and hormonal therapy) with consistent follow-up. The standardized assessment of LUTSs using validated tools such as the International Consultation on Incontinence Questionnaire (ICIQ), Overactive Bladder Symptom Score (OABSS), or American Urological Association Symptom Index (AUASI) should be implemented at baseline and follow-up to improve comparability across studies. Stratification by fibroid characteristics (e.g., size and position), hormonal status, and comorbidities should also be considered to identify patient subgroups that may benefit most from specific interventions.

## 5. Conclusions

In conclusion, this systematic review and meta-analysis shed light on the significant prevalence and impact of LUTSs in individuals with uterine myoma. The findings underscore the importance of the comprehensive evaluation and management of urinary symptoms in UM patients, considering factors such as BMI and subtype-specific manifestations of LUTSs. Further research is warranted to elucidate the underlying mechanisms and optimize management strategies for LUTSs in this population.

## Figures and Tables

**Table 1:** Univariate random-effects meta-regression for the prevalence of LUTSs according to age categories, parity, BMI, WHO regions, and risk of bias.

Variables		Number of Studies	Coefficient (95% CI)	Prevalence (95% CI)	*p*-Value	I^2^ (%)
By age		19	−0.09 (−0.52, 0.33)	0.49 (0.26, 0.72)	0.67	99.82
By parity		6	0.58 (−0.69, 1.84)	0.50 (0.35, 0.65)	0.37	99.17
By BMI		9	0.51 (0.29, 0.73)	0.63 (0.49, 0.75)	<0.001	96.51
WHO regions	European	10	Ref	0.53 (0.42, 0.64)	Ref	96.57
	Americas	8	0.15 (−0.45, 0.74)	0.56 (0.48, 0.64)	0.62	
	Western Pacific	1	−5.85 (−7.01, −4.61)	0.003 (0.003, 0.004)	<0.001	
	Eastern Mediterranean	-	-	-	-	
	South-East Asia	-	-	-	-	
	African	-	-	-	-	
Risk of bias	Low	17	Ref	0.48 (0.24, 0.74)	Ref	99.81
	Some concerns	3	0.20 (−2.56, 2.95)	0.53 (0.23, 0.80)	0.89	
	High	-	-	-	-	

**Abbreviations**: LUTS, lower urinary tract symptoms; BMI, body mass index; WHO, World Health Organization.

## Supplementary Materials

The following supporting information can be downloaded at https://www.mdpi.com/article/10.3390/medicina61050890/s1. Figure S1: Risk assessment of bias for included studies; Figure S2: The pooled prevalence of LUTS by subtype among patients with UM without hysterectomy; Figure S3: The pooled prevalence of LUTS by subtype among patients with UM with hysterectomy; Figure S4: The pooled odds ratio of SUI and UUI when comparing the UM arm to the non-UM arm; Figure S5: The pooled standardized mean difference of UM size across all subtypes of LUTS; Figure S6: The pooled odds ratio of LUTS comparing the non-hysterectomy arm to the post-hysterectomy arm; Table S1: Detail searching strategies for PubMed/Medline, Embase from January 2000 to September 2023; Table S2: Studies evaluating the prevalence of lower urinary tract symptoms (LUTS) among patients with uterine myoma (UM); Table S3: This table summarizes the 10-item risk of bias assessment tool adapted from Hoy et al. (2012), used to evaluate the methodological quality of prevalence studies included in this systematic review;
