# Supplementary material for: Lower Urinary Tract Symptoms in Uterine Myoma: A Systematic Review and Meta-Analysis
Source: Medicina (Kaunas). 2025 May 14;61(5):890. doi: 10.3390/medicina61050890 (PMC12112955; doi:10.3390/medicina61050890)
Supplement: Supplementary file 1 [file medicina-61-00890-s001.zip › medicina-3582687-supplementary.docx]

**Supplementary materials**

**Table S1.** Detail searching strategies for PubMed/Medline, Embase from January 2000 to September 2023.

**Pubmed/Medline**

| **Concept #1. Uterine myoma 35925** |
| --- |
| "Myoma"[MeSH Terms] OR "Leiomyoma"[MeSH Terms] OR "Myoma*"[TIAB] OR "Uterine myoma*"[TIAB] OR "Leiomyoma*"[TIAB] OR "Fibroid Tumor*"[TIAB] OR "Fibromyoma*"[TIAB] OR "Fibroid*"[TIAB] OR "Fibroid Uterus"[TIAB] OR "Uterine Fibroma*"[TIAB] OR "Uterine Fibroid*"[TIAB] OR "Uterine leiomyoma*"[TIAB] |
| **Concept #2. Lower Urinary Tract Symptoms 153746** |
| "Lower Urinary Tract Symptoms"[MeSH Terms] OR "Dysuria"[MeSH Terms] OR "Urination Disorders"[MeSH Terms] OR "Urinary Retention"[MeSH Terms] OR "Nocturia"[MeSH Terms] OR "Urinary Bladder, Overactive"[MeSH Terms] OR "Urinary Bladder, Underactive"[MeSH Terms] OR "Urinary Incontinence"[MeSH Terms] OR "Urinary Incontinence, Stress"[MeSH Terms] OR "Urinary Incontinence, Urge"[MeSH Terms] OR "Lower Urinary Tract Symptom*"[TIAB] OR "Dysuria"[TIAB] OR "Urination Disorder*"[TIAB] OR "Urinary Retention"[TIAB] OR "Nocturia"[TIAB] OR "Nycturia"[TIAB] OR "Overactive Bladder*"[TIAB] OR "Overactive Urinary Bladder*"[TIAB] OR "Overactive Detrusor"[TIAB] OR "Overactive Detrusor Function*"[TIAB] OR "Detrusor Underactivity"[TIAB] OR "Underactive Urinary Bladder*"[TIAB] OR "Underactive Bladder*"[TIAB] OR "Underactive Detrusor*"[TIAB] OR "Underactive Detrusor Function*"[TIAB] OR "Hypotonic Bladder*"[TIAB] OR "Urinary Incontinence"[TIAB] OR "Urinary Stress Incontinence"[TIAB] OR "Stress Incontinence"[TIAB] OR "Urinary Urge Incontinence"[TIAB] OR "Urge Incontinence"[TIAB] |
| **Concept #3: Restrict to human study 9521343** |
| {"animals"[MeSH Terms] NOT ("humans"[MeSH Terms] AND "animals"[MeSH Terms])} OR plant OR spacecraft OR worm* OR phonon OR *worm OR rat* OR mice OR rodents OR mouse OR dog OR cat OR bovine OR plant OR microorganism OR worm OR marine OR mammal* OR ocean OR wavelength OR spacecraft OR voltammeter* OR phonon OR worm* OR *worm OR magnetite OR sequencing   \| **Concept #4: Time 22662342** \| \| --- \| \| ("2000/01/01"[PDat]: "2023/09/24"[PDat]) \| |
| **Strategy 283** |
| (#1 AND #2 AND #4) NOT #3 |

**Embase**

| **Concept #1. Uterine myoma 41142** |
| --- |
| 'myoma*':ti,ab OR 'uterine myoma*':ti,ab OR 'leiomyoma*':ti,ab OR 'fibroid tumor*':ti,ab OR 'fibromyoma*':ti,ab OR 'fibroid*':ti,ab OR 'fibroid uterus':ti,ab OR 'uterine fibroma*':ti,ab OR 'uterine fibroid*':ti,ab OR 'uterine leiomyoma*':ti,ab |
| **Concept #2. Lower Urinary Tract Symptoms 102231** |
| 'lower urinary tract symptom*':ti,ab OR 'dysuria':ti,ab OR 'urination disorder*':ti,ab OR 'urinary retention':ti,ab OR 'nocturia':ti,ab OR 'nycturia':ti,ab OR 'overactive bladder*':ti,ab OR 'overactive urinary bladder*':ti,ab OR 'overactive detrusor':ti,ab OR 'overactive detrusor function*':ti,ab OR 'detrusor underactivity':ti,ab OR 'underactive urinary bladder*':ti,ab OR 'underactive bladder*':ti,ab OR 'underactive detrusor*':ti,ab OR 'underactive detrusor function*':ti,ab OR 'hypotonic bladder*':ti,ab OR 'urinary incontinence':ti,ab OR 'urinary stress incontinence':ti,ab OR 'stress incontinence':ti,ab OR 'urinary urge incontinence':ti,ab OR 'urge incontinence':ti,ab |
| **Concept #3: Restrict to human study, Clinical Study and EMBASE results 10220008** |
| [humans]/lim AND [clinical study]/lim AND [embase]/lim   \| **Concept #4: Time 29146899** \| \| --- \| \| [1-1-2000]/sd NOT [25-9-2023]/sd AND [1990-2023]/py \| |
| **Strategy 289** |
| #1 AND #2 AND #3 AND #4 |

**Table S2.** Studies evaluating the prevalence of lower urinary tract symptoms (LUTS) among patients with uterine myoma (UM).

| Author | Year | Age | Age standard deviation | Country | WHO regions | Risk of bias | No. of patient with UM | Prevalence of LUTS |
| --- | --- | --- | --- | --- | --- | --- | --- | --- |
| Nevadunsky, N. S., et al. (2001) ^1^ | 2001 | 44 | 7.3 | USA | Region of the Americas | Low | 84 | 28.57% |
| Pron, G., et al. (2003)^2^ | 2003 | 43 | 9.3 | Canada | Region of the Americas | Low | 538 | 56.88% |
| Audonnet, G. M., et al. (2004)^3^ | 2004 | 47.8 | 9.5 | France | European Region | Low | 30 | 93.33% |
| Waetjen, L. E., et al. (2007)^4^ | 2007 | 45.8 | 2.7 | USA | Region of the Americas | Low | 546 | 64.47% |
| Dragomir, A. D., et al. (2010)^5^ | 2010 | 45.8 | 3.9 | USA | Region of the Americas | Low | 534 | 51.31% |
| Parker-Autry, C., et al. (2011)^6^ | 2011 | 42.4 | 7.6 | USA | Region of the Americas | Low | 78 | 91.03% |
| Ruuskanen, A. J., et al. (2012)^7^ | 2012 | 47.5 | 5.5 | Finland | European Region | Low | 122 | 55.74% |
| García-Pérez, H., et al. (2013)^8^ | 2013 | 39.5 | 10 | Mexico | Region of the Americas | Low | 79 | 34.17% |
| Shveiky, D., et al. (2013)^9^ | 2013 | 45 | 2 | Isreal | European Region | Low | 57 | 85.96% |
| Ekin, M., et al. (2014)^10^ | 2014 | 43 | 6.3 | Turkey | European Region | Low | 155 | 76.77% |
| Vecchioli-Scaldazza, C., et al. (2016)^11^ | 2016 | 44.1 | 7.2 | Italy | European Region | Some concerns | 44 | 72.73% |
| Bochenska, K., et al. (2017)^12^ | 2017 | 41 | 6 | USA | Region of the Americas | Some concerns | 195 | 64.10% |
| Pålsson, M., et al. (2017)^13^ | 2017 | 45.6 | 3.1 | Sweden | European Region | Low | 3850 | 22.70% |
| Bohlin, K. S., et al. (2017)^14^ | 2017 | 50.7 | 17.1 | Sweden | European Region | Low | 2881 | 31.38% |
| Mohr-Sasson, A., et al. (2018)^15^ | 2018 | 41.1 | 3.8 | Isreal | European Region | Some concerns | 132 | 23.48% |
| Shaffer, R. K., et al. (2019)^16^ | 2019 | 43 | 6.4 | USA | Region of the Americas | Low | 338 | 52.96% |
| Berujon, E., et al. (2022)^17^ | 2022 | 43.3 | 7.2 | France | European Region | Low | 55 | 63.64% |
| Shin, J. H., et al. (2022)^18^ | 2022 | 44.1 | 7.2 | Korea | Western Pacific Region | Low | 160 (post-hysterectomy) | 70.63% (post-hysterectomy) |
| Proshchenko, O. M. and I. B. Ventskivska (2022)^19^ | 2022 | 46.3 | 1.5 | Ukraine | European Region | Low | 50 | 22.00% |
| Yuk, J. S. and J. H. Lee (2023)^20^ | 2023 | 46.5 | 1.5 | Korea | Western Pacific Region | Low | 116390 | 0.32% |

**Table S3.** This table summarizes the 10-item risk of bias assessment tool adapted from Hoy et al. (2012), used to evaluate the methodological quality of prevalence studies included in this systematic review.

| **Domain** | **Item** | **Description** |
| --- | --- | --- |
| **External Validity** | **1** | **Was the study’s target population a close representation of the national population in relation to relevant variables?** |
| **External Validity** | **2** | **Was the sampling frame a true or close representation of the target population?** |
| **External Validity** | **3** | **Was some form of random selection used to select the sample, or was a census undertaken?** |
| **External Validity** | **4** | **Was the likelihood of nonresponse bias minimal?** |
| **Internal Validity – Measurement Bias** | **5** | **Were data collected directly from the subjects (as opposed to a proxy)?** |
| **Internal Validity – Measurement Bias** | **6** | **Was an acceptable case definition used in the study?** |
| **Internal Validity – Measurement Bias** | **7** | **Was the study instrument that measured the parameter of interest shown to have validity and reliability?** |
| **Internal Validity – Measurement Bias** | **8** | **Was the same mode of data collection used for all subjects?** |
| **Internal Validity – Measurement Bias** | **9** | **Was the length of the shortest prevalence period for the parameter of interest appropriate?** |
| **Internal Validity – Analysis Bias** | **10** | **Were the numerator(s) and denominator(s) for the parameter of interest appropriate?** |

**Figure S1.** Risk assessment of bias for included studies

a.


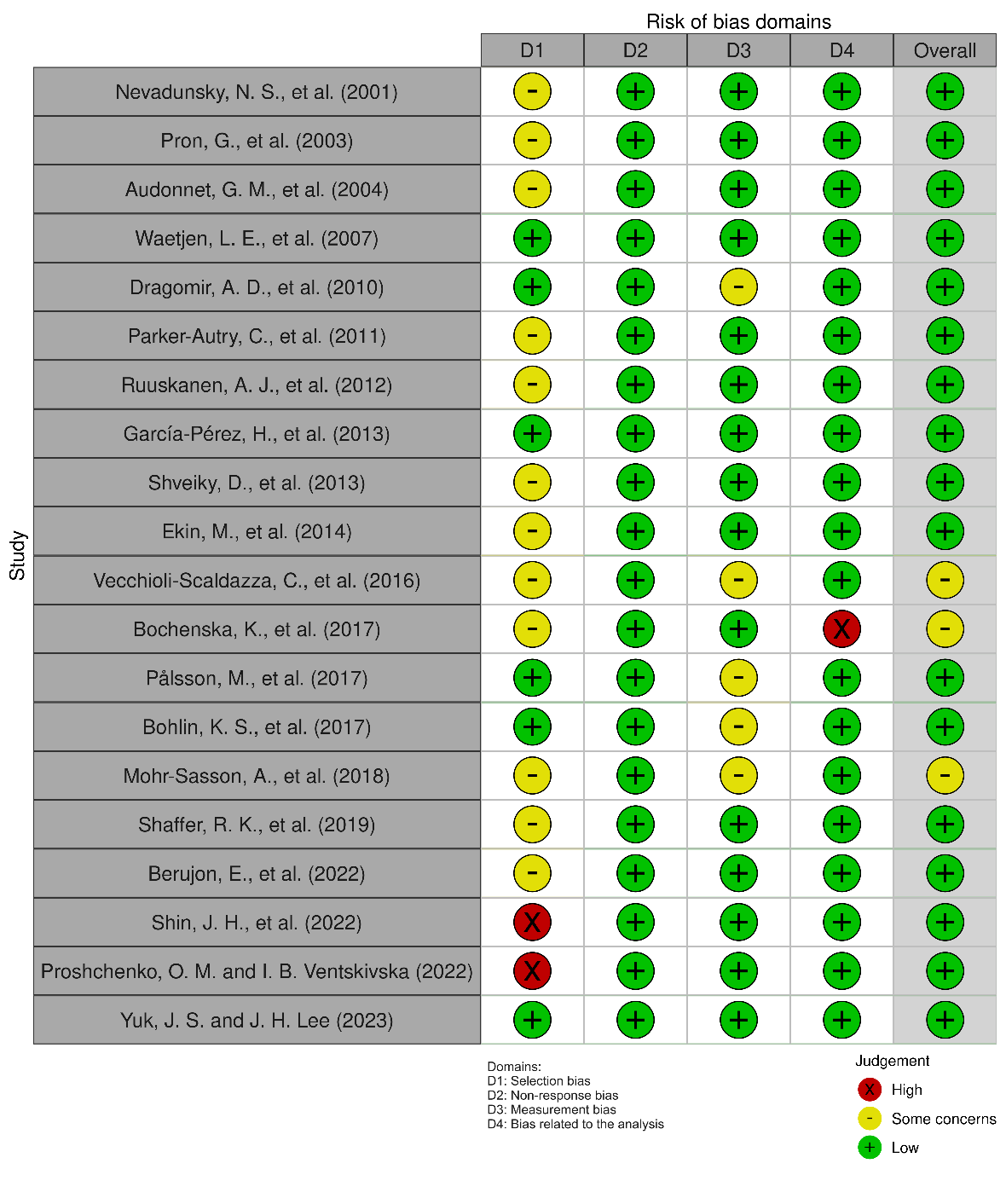


b.


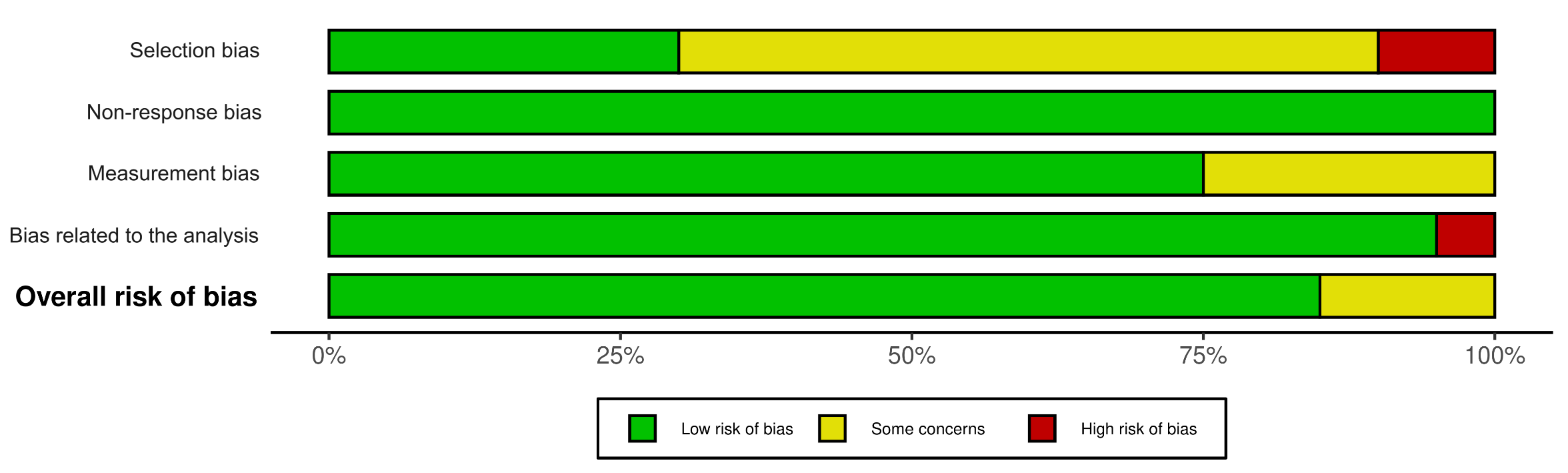


**Figure S2.** The pooled prevalence of LUTS by subtype among patients with UM without hysterectomy.

a. Urinary Frequency


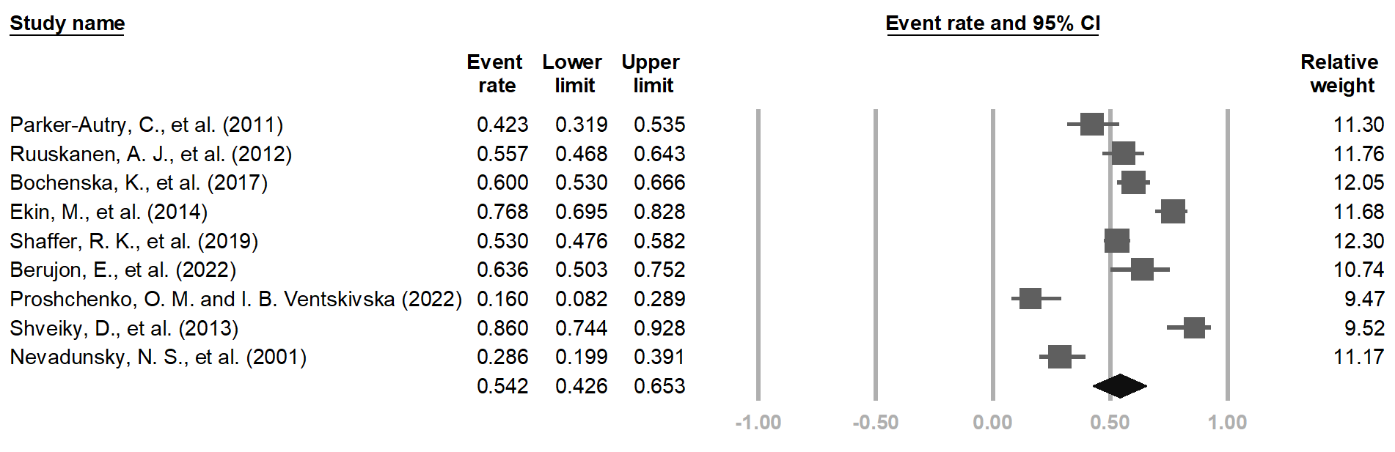


b. Urinary Urgency


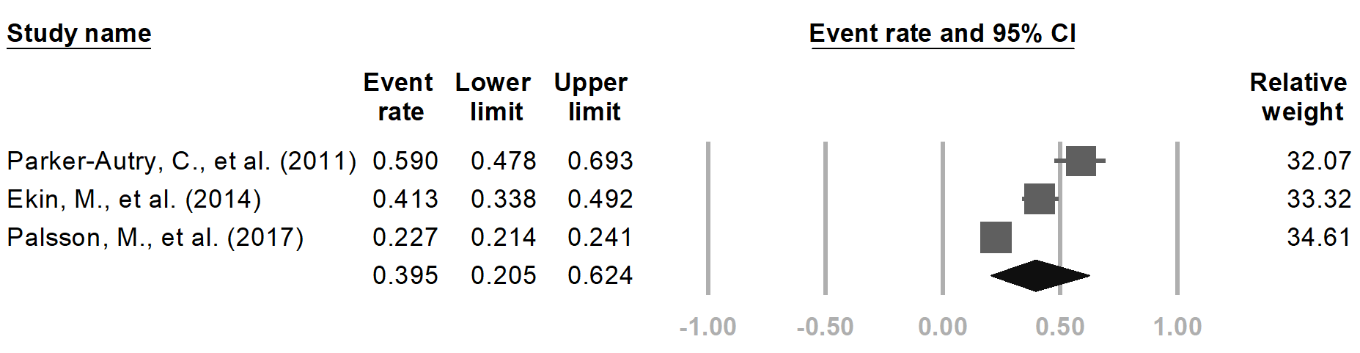


c. Nocturia


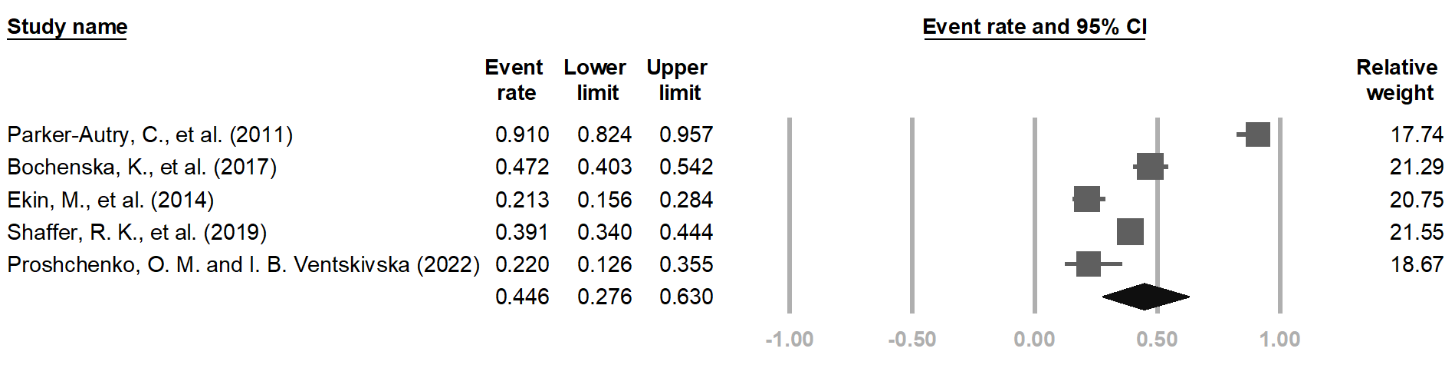


d. Urinary Incontinence


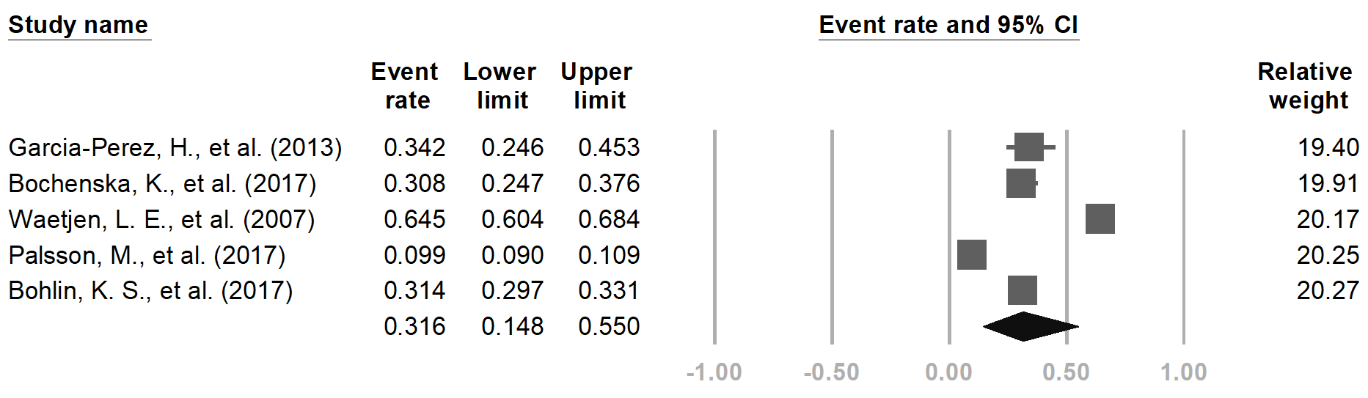


e. Stress Urinary Incontinence


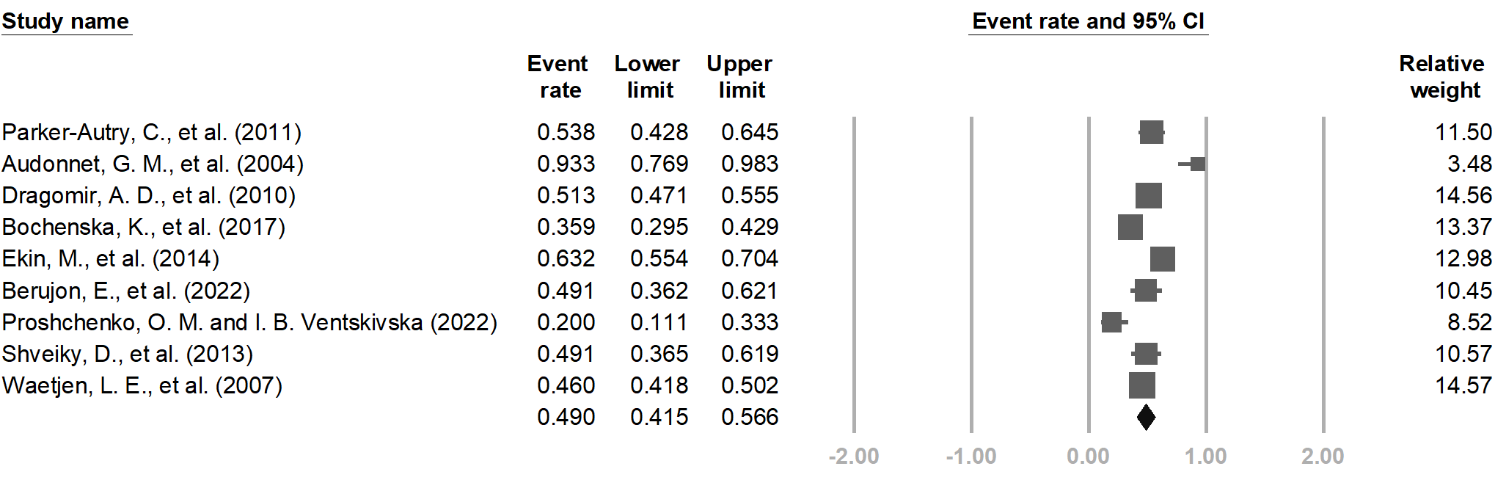


f. Urgency Urinary Incontinence


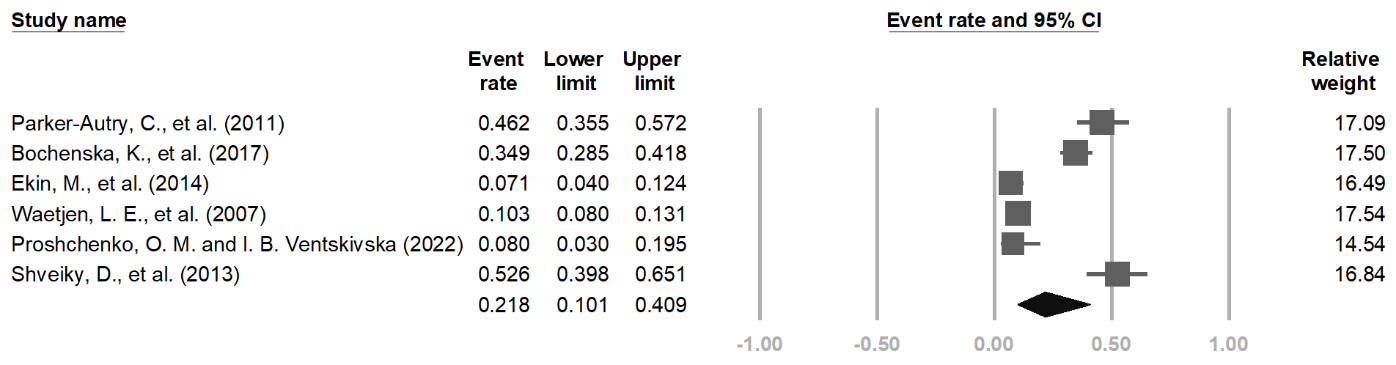


g. Mixed Urinary Incontinence


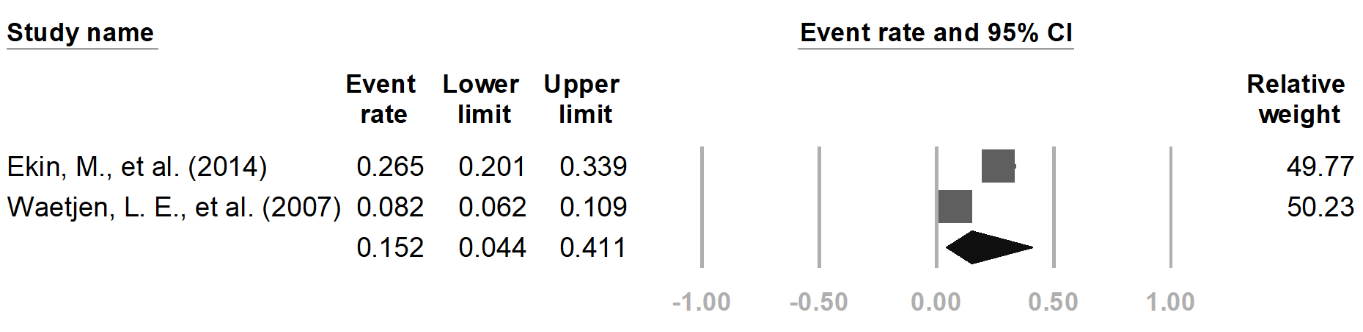


h. Voiding Symptoms


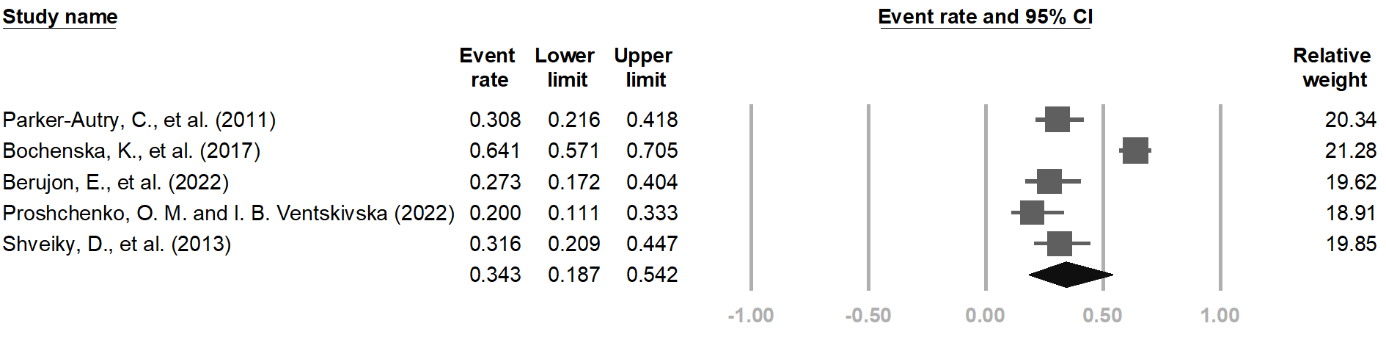


i. Overactive Bladder


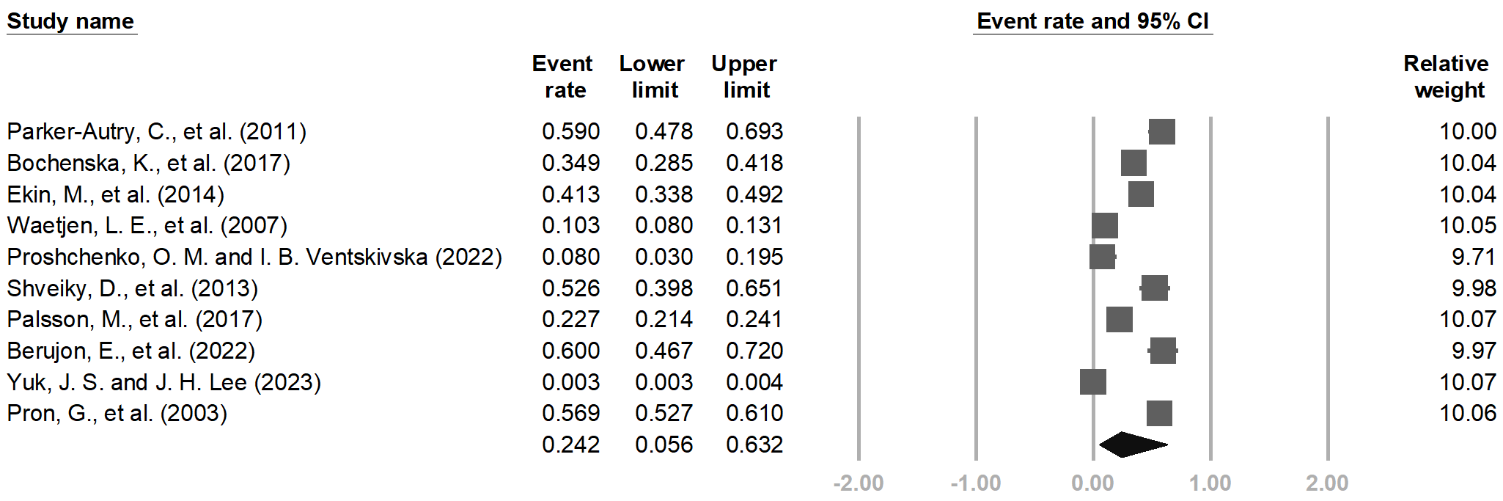


**Figure S3.** The pooled prevalence of LUTS by subtype among patients with UM with hysterectomy.

a. Urinary Frequency


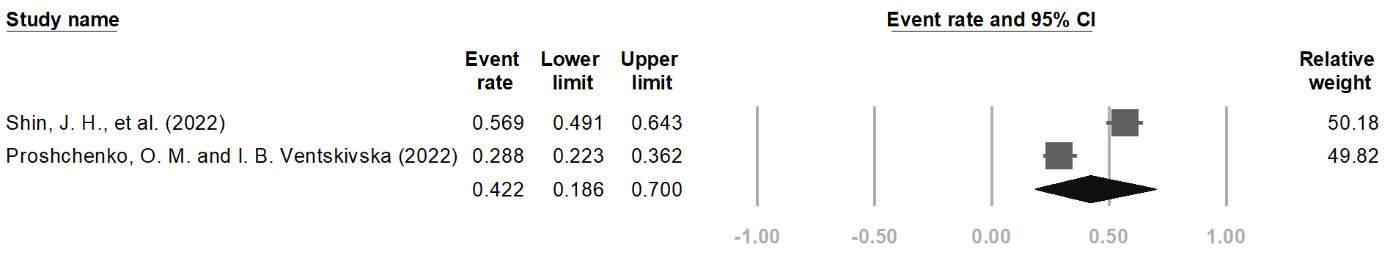


b. Urinary Urgency


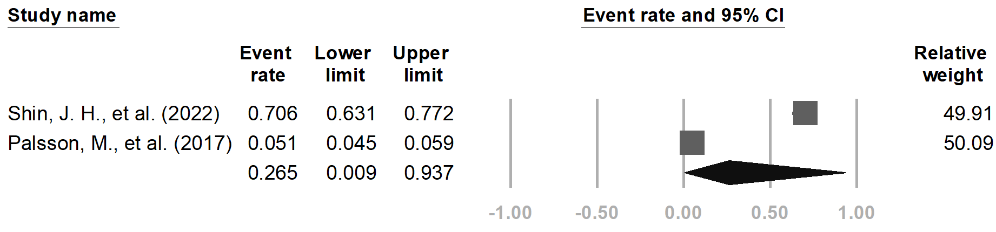


c. Urinary Incontinence


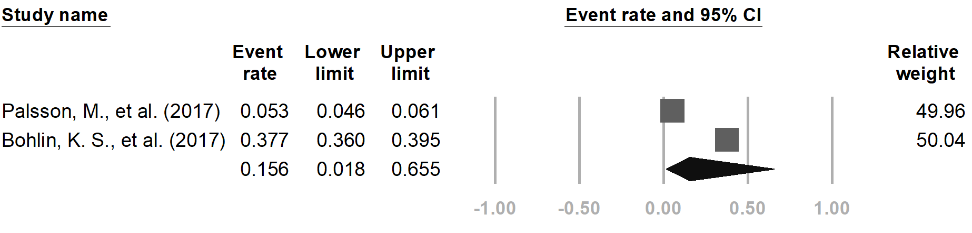


d. Stress Urinary Incontinence


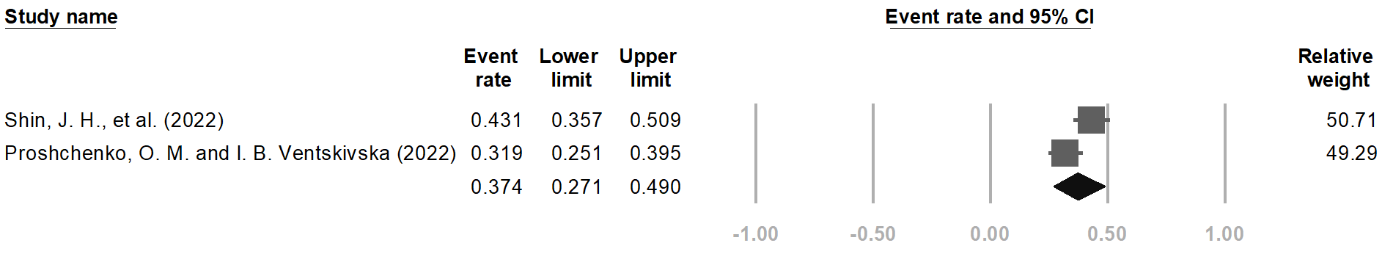


e. Urgency Urinary Incontinence


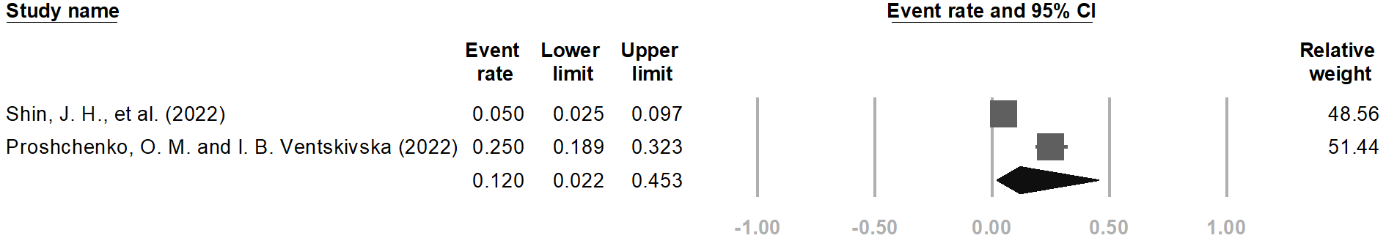


f. Voiding Symptoms


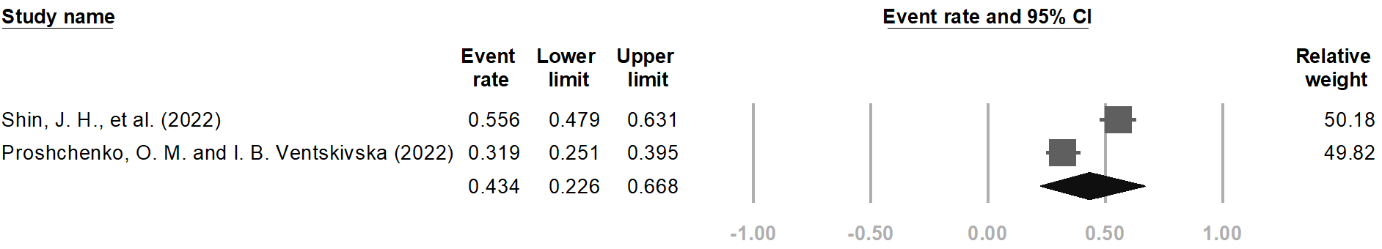


**Figure S4.** The pooled odds ratio of SUI and UUI when comparing the UM arm to the non-UM arm.

a. SUI


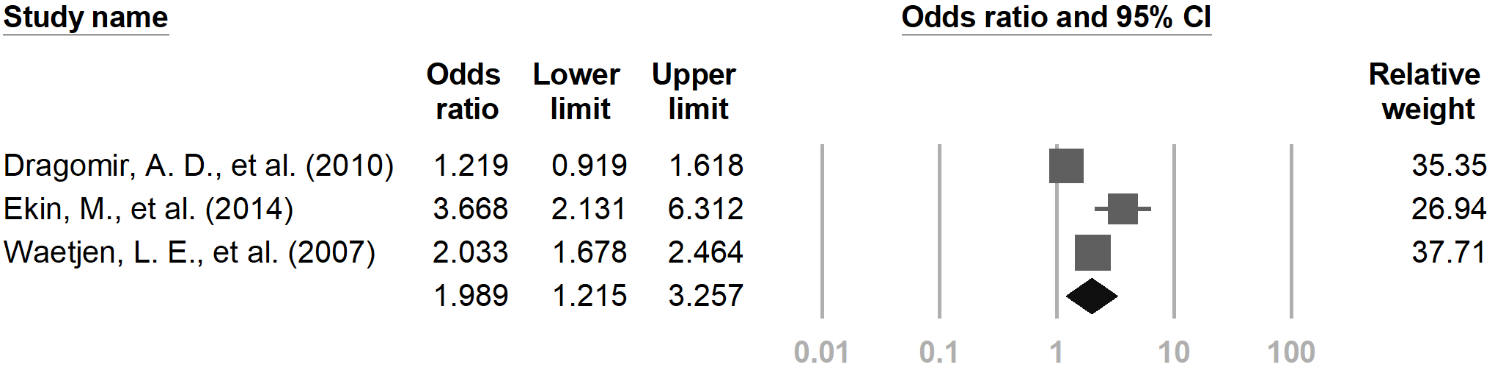


b. UUI


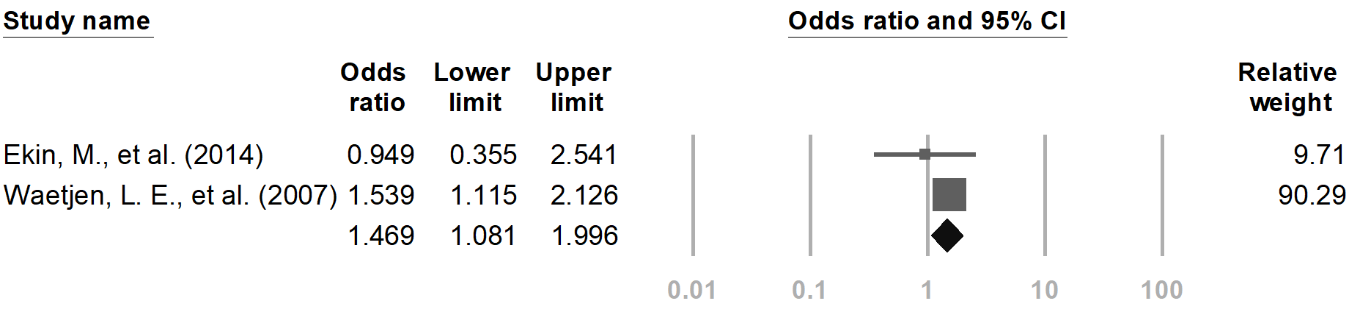


c. MUI


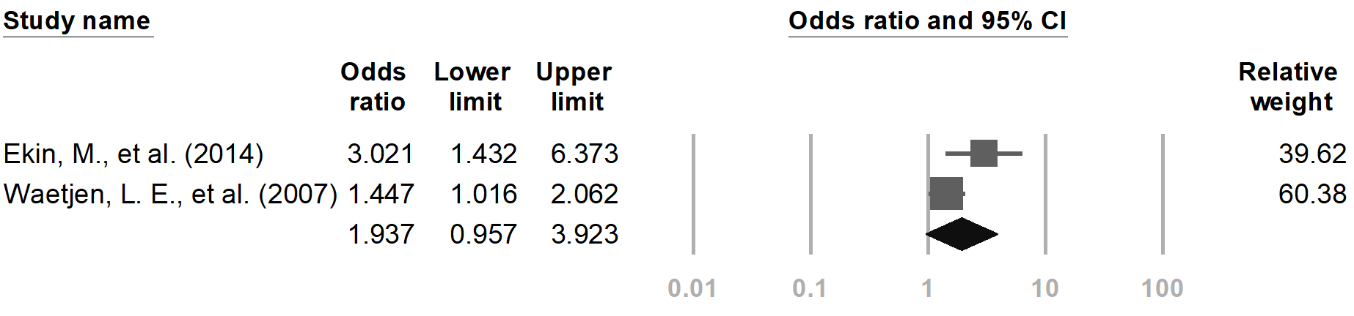


**Figure S5.** The pooled standardized mean difference of UM size across all subtypes of LUTS.

a. Urinary Frequency


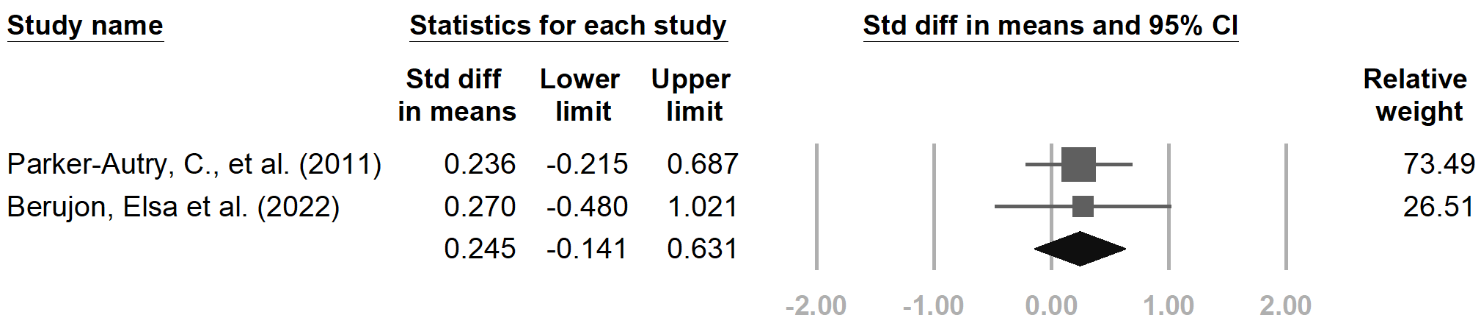


b. SUI


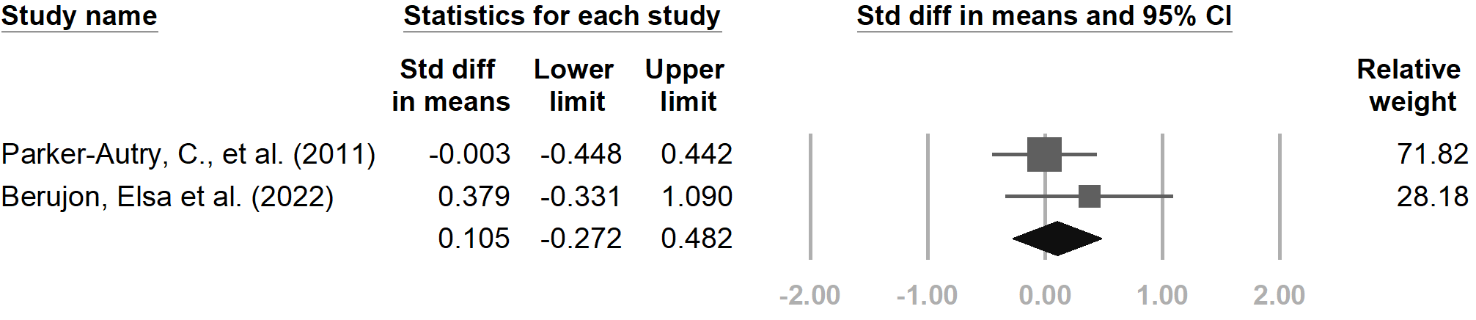


c. UUI


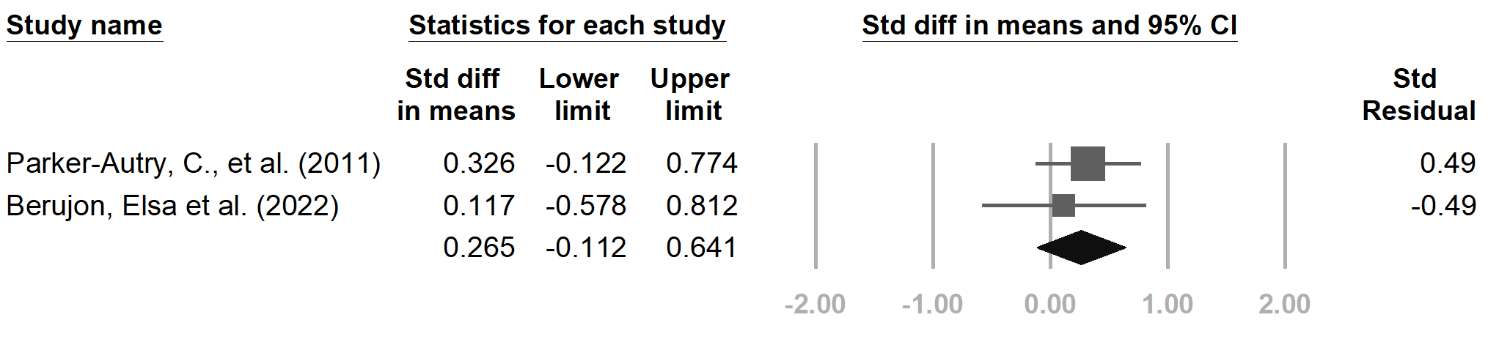


d. Voiding Symptoms


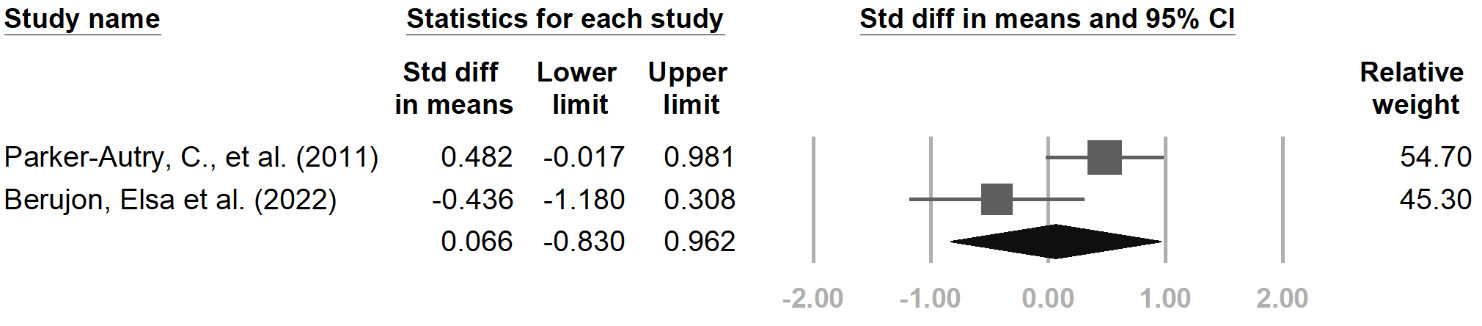


**Figure S6.** The pooled odds ratio of LUTS comparing the non-hysterectomy arm to the post-hysterectomy arm.


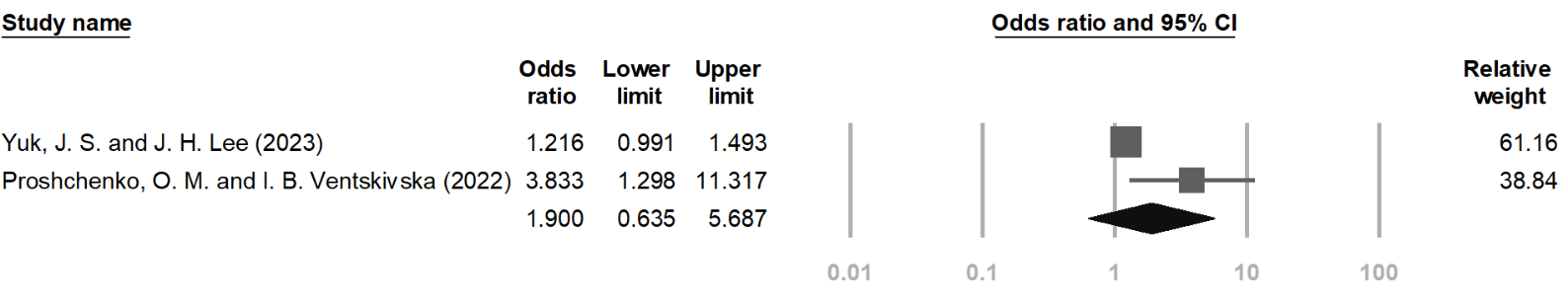


**References**

1. Nevadunsky NS, Bachmann GA, Nosher J, Yu T. Women's decision-making determinants in choosing uterine artery embolization for symptomatic fibroids. *The Journal of reproductive medicine* 2001; **46**(10): 870-4.

2. Pron G, Bennett J, Common A, Wall J, Asch M, Sniderman K. The Ontario Uterine Fibroid Embolization Trial. Part 2. Uterine fibroid reduction and symptom relief after uterine artery embolization for fibroids. *Fertility and sterility* 2003; **79**(1): 120-7.

3. Audonnet GM, Valentini FA, Nelson PP, Breheret J. Urodynamics and uterine fibroid. Is there a specific parameter? *Annales de Readaptation et de Medecine Physique* 2004; **47**(2): 51-5.

4. Waetjen LE, Liao S, Johnson WO, et al. Factors associated with prevalent and incident urinary incontinence in a cohort of midlife women: A longitudinal analysis of data: Study of women's health across the nation. *American journal of epidemiology* 2007; **165**(3): 309-18.

5. Dragomir AD, Schroeder JC, Connolly A, et al. Uterine leiomyomata associated with self-reported stress urinary incontinence. *Journal of women's health (2002)* 2010; **19**(2): 245-50.

6. Parker-Autry C, Harvie H, Arya LA, Northington GM. Lower urinary tract symptoms in patients with uterine fibroids: association with fibroid location and uterine volume. *Female pelvic medicine & reconstructive surgery* 2011; **17**(2): 91-6.

7. Ruuskanen AJ, Hippeläinen MI, Sipola P, Manninen HI. Association between magnetic resonance imaging findings of uterine leiomyomas and symptoms demanding treatment. *European journal of radiology* 2012; **81**(8): 1957-64.

8. García-Pérez H, Harlow SD, Sampselle CM, Denman C. Measuring urinary incontinence in a population of women in northern Mexico: Prevalence and severity. *International urogynecology journal and pelvic floor dysfunction* 2013; **24**(5): 847-54.

9. Shveiky D, Iglesia CB, Antosh DD, et al. The effect of uterine fibroid embolization on lower urinary tract symptoms. *International urogynecology journal and pelvic floor dysfunction* 2013; **24**(8): 1341-5.

10. Ekin M, Cengiz H, Öztürk E, Kaya C, Yasar L, Savan K. Genitourinary symptoms and their effects on quality of life in women with uterine myomas. *International urogynecology journal and pelvic floor dysfunction* 2014; **25**(6): 807-10.

11. Vecchioli-Scaldazza C, Morosetti C, Vichi M, Bini E, Giannubilo W, Ferrara V. Effect of Surgical Therapy on Urinary Symptoms and Urodynamic Findings in Patients with Uterine Myoma. *The Journal of reproductive medicine* 2016; **61**(9-10): 436-40.

12. Bochenska K, LeWitt T, Marsh EE, et al. Fibroids and urinary symptoms study (FUSS). *American journal of obstetrics and gynecology* 2017; **216**(3): S617.

13. Pålsson M, Stjerndahl JH, Granåsen G, Löfgren M, Sundfeldt K. Patient-reported lower urinary tract symptoms after hysterectomy or hysteroscopy: a study from the Swedish Quality Register for Gynecological Surgery. *International urogynecology journal* 2017; **28**(9): 1341-9.

14. Bohlin KS, Ankardal M, Lindkvist H, Milsom I. Factors influencing the incidence and remission of urinary incontinence after hysterectomy. *American journal of obstetrics and gynecology* 2017; **216**(1): 53.e1-.e9.

15. Mohr-Sasson A, Machtinger R, Mashiach R, et al. Long-term outcome of MR-guided focused ultrasound treatment and laparoscopic myomectomy for symptomatic uterine fibroid tumors. *American journal of obstetrics and gynecology* 2018; **219**(4): 375.e1-.e7.

16. Shaffer RK, Dobberfuhl AD, Vu KN, et al. Are fibroid and bony pelvis characteristics associated with urinary and pelvic symptom severity? *American journal of obstetrics and gynecology* 2019; **220**(5): 471.e1-.e11.

17. Berujon E, Thubert T, Fauvet R, Villot A, Pizzoferrato AC. Impact of uterine fibroid surgery on lower urinary tract symptoms. *Journal of gynecology obstetrics and human reproduction* 2022; **51**(5).

18. Shin JH, Gwak CH, Park MU, Choo MS. Effects of different types of hysterectomies on postoperative urodynamics and lower urinary tract symptoms. *Investigative and clinical urology* 2022; **63**(2): 207-13.

19. Proshchenko OM, Ventskivska IB. EFFICIENCY OF TREATMENT AND DIAGNOSTIC ALGORITHMS IN THE REHABILITATION PROGRAM OF WOMEN AFTER HYSTERECTOMY WITH OPPORTUNIST SALPINGECTOMY DUE TO UTERINE MYOMA. *Reproductive Endocrinology* 2022; (66): 90-7.

20. Yuk JS, Lee JH. Risk of overactive bladder after hysterectomy for uterine fibroids. *International urogynecology journal* 2023; **34**(8): 1823-9.
